# Kisspeptin expression levels in patients with placenta previa: A randomized trial

**DOI:** 10.1097/MD.0000000000038866

**Published:** 2024-07-12

**Authors:** Yunus Katirci, Adem Kocaman, Ayse Zehra Ozdemir

**Affiliations:** a Department of Gynecology and Obstetrics, Faculty of Medicine, Ondokuz Mayis University, Samsun, Turkey; b Department of Histology and Embryology, Faculty of Medicine, Ondokuz Mayis University, Samsun, Türkiye.

**Keywords:** fetal ultrasonography, kisspeptin, placenta previa

## Abstract

**Background:**

This study aimed to explore the potential influence of kisspeptin (KISS1) levels on the etiology of placenta previa for early pregnancy diagnosis.

**Methods:**

The study included 20 pregnant women diagnosed with placenta previa and 20 pregnant woman with normal pregnancies between 2021 and 2022. Plasma KISS1 levels were determined through biochemical analysis, while genetic analysis assessed KISS1 and KISS1 receptor gene expression levels. Immunohistochemical methods were employed to determine placenta KISS1 levels.

**Results:**

The evaluation of KISS1 concentration in serum revealed a significant decrease in the placenta previa group compared to the control group (*P* < .001). KISS1 gene expression level 0.043-fold decreased in the placenta previa group (*P* < .001). Furthermore, the KISS1 receptor gene expression level increased 170-fold in the placenta previa group.

**Conclusions:**

Results from biochemical, immunohistochemical, and genetic analyses consistently indicated significantly reduced KISS1 expression in patients with placenta previa. These findings suggest a potential link between diminished KISS1 levels and the occurrence of placenta previa. KISS1 may play a critical role in the etiology of placenta previa. Detailed studies on angiogenesis, cell migration and tissue modeling should be conducted to understand possible mechanisms.

## 1. Introduction

Placenta development is critical for pregnancy. As the largest fetal organ, it has extraordinary functions in the development and protection of the fetus.^[1]^ Abnormalities related to the location and anatomy of the placenta during pregnancy may occur. These include placentas localized in the lower region, placenta previa, and abnormally invasive placentas.^[2]^ These conditions pose a risk of antepartum, intrapartum, and postpartum bleeding. Additionally, it may affect placental functions and maternal or fetal health status.^[3,4]^ The incidence is increasing, predominantly due to the elevated cesarean section rate.^[2,5,6]^ Other factors affecting the placenta include previous uterine surgeries or curettage, maternal age, and multiparity.^[2]^ In addition, the incidence of placenta previa is also increasing due to endometriosis, smoking and assisted reproductive technology.^[7,8]^ Treatment options for this condition are few and pregnancy is usually concluded with a cesarean section. Unfortunately, the diagnosis of placenta previa can often be detected in the very advanced weeks of pregnancy. Late diagnosis may cause pregnant women to die due to bleeding. At this point, detecting a protein that may be associated with placenta previa in the early stages of pregnancy through routine biochemical or genetic analysis is of vital importance in monitoring this disease, keeping the pregnancy under control, and being prepared for possible situations.

Data from animal and human studies indicate that various regulatory molecules play functional roles in the control of trophoblast invasion and placental angiogenesis. Normal trophoblast invasion and migration are crucial for the development of the placenta. Disruption of these processes from the early stages leads to ischemia and a decrease in uteroplacental flow that harms the structure and function of the placenta. At this point, kisspeptin (KISS1), which has been associated with implantation and placenta development in recent years, attracts attention. KISS1 is produced by trophoblasts. As trophoblasts begin to invade, plasma KISS1 concentrations during the peri-implantation period may reflect early developmental events associated with pregnancy outcome.^[9]^ In humans, KISS1 plasma concentration increases markedly during pregnancy.^[10]^ The possible effects of KISS1 and kisspeptin receptor (KISS1R) on intracellular skeletal organization, cell migration and collagenase activity have been reported in previous studies.^[11,12]^ The migration of placental cells and intercellular collagenase activity may play an important role in patients who develop placenta previa. At this point, KISS1 and KISS1R levels during placental development may play a critical role in abnormal placental development.

The etiology of placenta previa is not fully understood. To date, adequate preventive strategies have not been developed other than reducing uterine surgery such as cesarean section, dilation and curettage, and avoiding unnecessary use of assisted reproductive techniques. Such pregnancies often result in the death of the mother or loss of the uterus. It is important to detect early findings that may indicate the development of placenta previa. This study aimed to elucidate the possible role of KISS1 in diagnosing placenta previa in the first 20 weeks of pregnancy by investigating KISS1 expression levels in plasma and placenta sample.

## 2. Materials and methods

### 2.1. Participants and material collection

Twenty pregnant women who were diagnosed with placenta previa via ultrasonography and met the inclusion criteria were included in the study as the experimental group. Twenty pregnant women who did not have any placental anomalies and had a healthy birth were included in the control group. This study was evaluated by the Ondokuz Mayis University Clinical Research Ethics Committee, and approved with the number of OMÜ KAEK 2021/481 board decision dated 12 November, 2021. The purpose of the study was fully explained to the patients and informed written consent was obtained from the patients. Blood samples were taken from patients in the second and third trimesters. After the pregnant women gave birth by cesarean section, the placenta tissue was removed. At the umbilical cord exit, tissue samples were taken along the entire thickness with systematic random sampling in 4 directions parallel to the placenta surface and at an equal distance from the umbilical cord. Half of the samples taken were placed in physiological saline for genetic analysis and stored in a deep freezer at ‐80 °C. The other part was transferred to containers containing 4% formaldehyde for histological analysis.

### 2.2. ELISA assay

Biochemical analysis of KISS1 in plasma samples were performed using the competitive-enzyme-linked immunosorbent assay (ELISA) method. The human KISS-1 ELISA kit (Elabscience, #E-EL-H5618) was used following the manufacturer’s protocol. The concentration of KISS1 protein in the range of 62.5 to 4000 pg/mL were measured in human plasma with a sample volume of 100 µL. Spectrophotometric measurement were carried out at 450 nm. With the pilot study, the dilution rates of plasma samples were determined and then all samples were tested using the microplate absorbance reader (Sunrise, Tecan Trading Ag, Mannedorf, Switzerland).

### 2.3. Quantitative real-time polymerase chain reaction (RT-qPCR)

Total ribonucleic acid (RNA) was extracted with the TRIzol (Invitrogen) according to the manufacturer’s instructions. RNA (10 μg) was reverse-transcribed into first-strand complementary deoxyribonucleic acid (cDNA) with the iScript Reverse Transcription Kit (Qiagen, Germany). Each 50 μL qPCR reaction contained 1X SYBR Green PCR Master Mix (Qiagen, Germany), 300 ng of cDNA, and 300 nM of each specific primer. The following primers were used: KISS1, 5′-CCACCTCTGGACATTCACC-3′ (sense) and 5′-GCTGCCAAGAAACCAGTGAG-3′ (antisense). RT-qPCR was performed on an Biorad CFX96 RT-qPCR detection system. The specificity of each assay was validated by melting curve analysis and agarose gel electrophoresis of the PCR products. All of the RT-qPCR experiments were run in duplicate, and a cycle threshold (Ct) value was used to determine the messenger RNA (mRNA) levels. Glyceraldehyde 3-phosphate dehydrogenase gene was used as the reference gene and relative quantification of the mRNA levels was performed using the comparative Ct method with the formula 2^–∆∆Ct^.

### 2.4. Statistical analysis

Statistical evaluations were carried out using the Prism 8 program. The results are presented as the mean ± standard error of the mean or mean ± standard deviation. The Shapiro–Wilk test is used for assessing whether a dataset follows a normal distribution. Demographic data and biochemical results showed normal distribution. The unpaired t test was used to evaluate demographic data and biochemical results. Gene expression results also showed normal distribution and unpaired t test was used to evaluate KISS1 and KISS1R Ct values. A significant difference was defined as *P* < .05 and a highly significant difference was defined as *P* < .01.

## 3. Results

### 3.1. Demographic data

The control group has a higher average number of pregnancies compared to the placenta previa group. The difference in the number of pregnancies between the 2 groups is statistically significant (*P* < .05) (Table 1).

**Table 1:** Data regarding the age, gravida, parity, and gestational weeks of the patients participating in the study.

	Control	Placenta previa	*P*
Age	31.74 ± 4.26	32.00 ± 4.89	0.56
Gravida	3.57 ± 1.64	2.52 ± 1.26	0.03*
Parity	2.15 ± 1.21	1.84 ± 1.16	0.41
Gestational weeks	34.37 ± 2.56	34.16 ± 2.71	0.80

Mean ± standard deviation.

* *P* < .05.

### 3.2. Biochemical analyses

**Figure 1.**
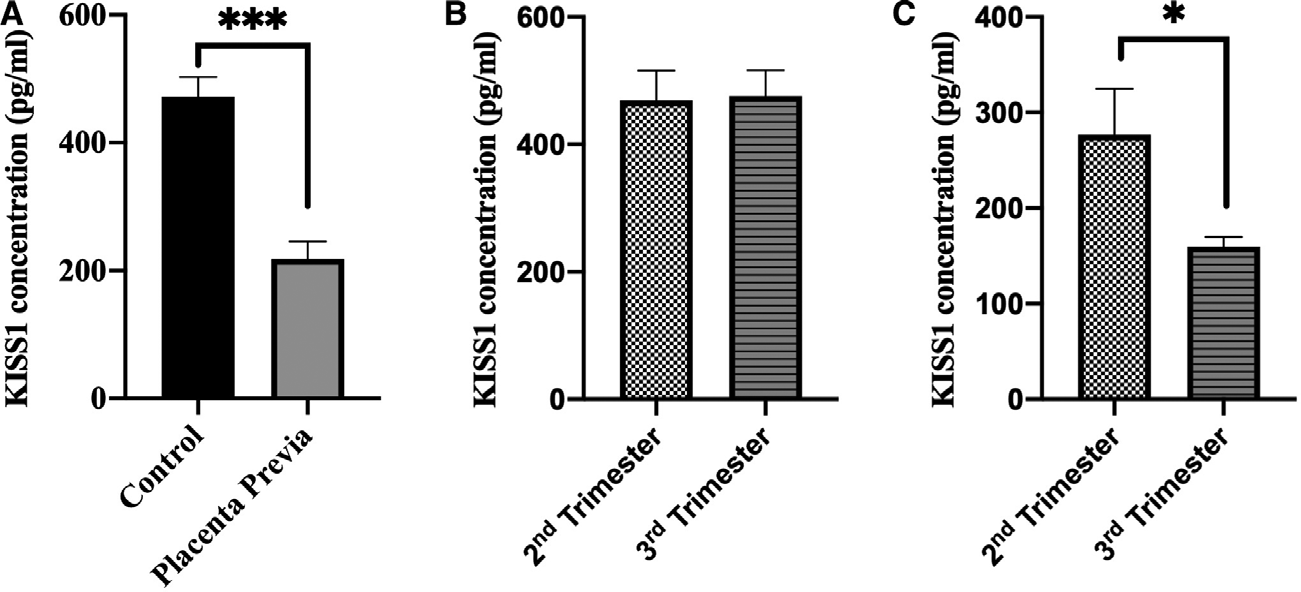
Graph A showing the difference in serum KISS1 level between control and placenta previa groups (mean ± SD), ***P < .001. Graph B showing the difference in serum KISS1 level between the 2nd and 3rd trimester in the control group (mean ± SD). Graph C showing the difference in serum KISS1 level between the 2nd and 3rd trimester in the placenta previa group (mean ± SD), *P < .05. KISS1 = kisspeptin.

As a result of the evaluation of KISS1 concentration level in serum, a significant decrease was observed in the placenta previa group compared to the control group (*P* < .001) (Fig. 1A).

The samples in the control and placenta previa group were categorized according to their gestational weeks and divided into 2 separate subcategories as the 2nd and 3rd trimester, and the KISS1 concentration level was evaluated. No statistically significant difference was detected between the subgroups in the control group (*P* = .795) (Fig. 1B). However, there was a significant decrease in the 3rd trimester of the placenta previa group compared to the 2nd trimester (*P < .05) (Fig. 1C).

### 3.3. Genetic analyses

**Figure 2.**
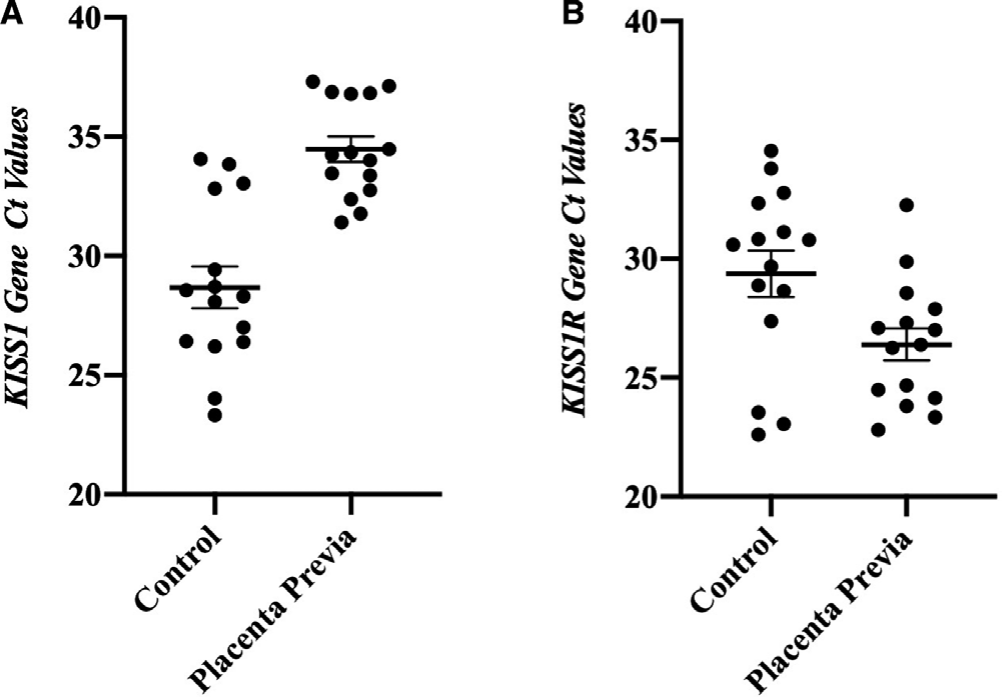
Ct value distribution of KISS1 and KISS1R genes. KISS1 = kisspeptin, KISS1R = kisspeptin receptor.

The KISS1 gene exhibited a normal distribution in average Ct values within both groups. A statistically significant difference was identified between the control and placenta previa groups (*P* < .001) (Fig. 2A).

The average Ct values for the KISS1R gene displayed a normal distribution in both groups. A statistically significant difference was observed between the control and placenta previa groups (*P* < .05) (Fig. 2B).

**Figure 3.**
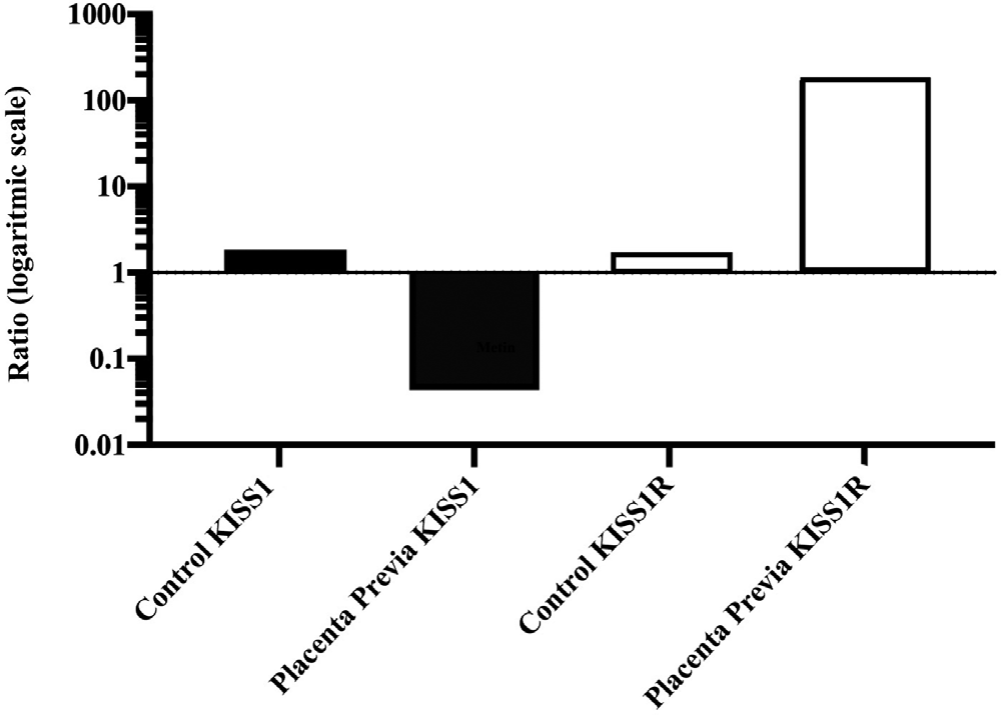
Comparison of KISS1 and KISS1R expression levels. KISS1 = kisspeptin, KISS1R = kisspeptin receptor.

Analysis of KISS1 gene expression revealed a substantial 0.043-fold reduction in the placenta previa group compared to the control group, demonstrating a highly significant statistical difference (*P* < .001). Examination of samples for KISS1R gene expression unveiled a notable 170-fold increase in the placenta previa group relative to the control group, with this discrepancy proving highly statistically significant (*P* < .001) (Fig. 3 and Table 2).

**Table 2:** Data regarding the KISS1 serum concentration, KISS1 and KISS1R gene expression levels.

	Control	Placenta previa	*P*
KISS1 serum concentration (pg/mL)	472.3 ± 135.3	218.4 ± 122.2	.01*
KISS1 gene CT values	28.68 ± 3.41	34.48 ± 2.04	.06*
KISS1R gene CT values	29.37 ± 3.78	26.40 ± 2.61	.02*
KISS1 fold change	1	0.043 (reduction)	
KISS1R fold change	1	170 (increase)	

Mean ± standard deviation.

* *P* < .05.

## 4. Discussion

In this study, a decrease in KISS1 gene expression levels was observed in patients with placenta previa, consistent with the biochemical data. Additionally, higher level of KISS1R expression was detected in the placenta previa group. Particularly, lower concentration of KISS1 was observed in the third trimester in the placenta previa group. These findings indicate the potential significance of KISS1 expression levels in the etiology of placenta previa.

Studies investigating the application of KISS1 have revealed its capacity to stimulate the release of gonadotropin-releasing hormone.^[13]^ In healthy premenopausal women, subcutaneous injection of KISS1 has been found to elevate plasma luteinizing hormone levels, particularly during the preovulatory phase of the menstrual cycle.^[14]^ Assessing the impact of KISS1 application in women with hypothalamic amenorrhea, Jayasena et al^[15,16]^ observed an initial increase in plasma gonadotropins with twice-daily subcutaneous administration. However, this effect diminished after 2 weeks. Interestingly, the same group of women exhibited a sustained gonadotropin response for 8 weeks when KISS1 was administered twice weekly.^[16]^ Furthermore, Park et al discovered an upregulation of KISS1 expression during decidualization in endometrial stromal cells, suggesting a potential role for KISS1 in preparing the endometrium for effective placentation.^[17]^

The expression of KISS1R in decidualized stromal cells is associated with the inhibition of cell motility, indirectly influencing placental invasion. Concurrently, placental KISS1 and its receptor, KISS1R, play a direct role in controlling placental invasion by constraining the migration and invasion of extra villous trophoblasts. The functional significance of the KISS1/KISS1R signaling systems in both the uterus and placenta suggests potential applications for KISS1. It could serve as a promising prognostic and diagnostic indicator for pregnancy-related conditions such as implantation failure, recurrent pregnancy loss, and preeclampsia. Furthermore, there is potential for KISS1 to be explored as a therapeutic agent in addressing these conditions.^[18]^

Numerous studies have explored serum KISS1 levels across diverse patient cohorts, particularly focusing on pregnant women in various trimesters and obstetric conditions. Research has indicated a significant rise in KISS1 plasma levels during pregnancy, with a 9000-fold increase in the first trimester and a 7000-fold increase in the third trimester.^[10]^ In our investigation, the control group exhibited consistent plasma KISS1 levels between the second and third trimesters. In contrast, patients with placenta previa showed a decline in KISS1 levels during the third trimester compared to the second trimester. These findings suggest a potential association between decreasing KISS1 levels as gestational weeks progress in placenta previa patients and an abnormal placentation process. While previous studies suggest no significant difference in KISS1 mRNA levels between early pregnancy and term placentas,^[19]^ Kisspeptin-54 levels have been notably higher in early-term placentas.^[20]^ This implies that KISS1 levels in the placenta may not precisely mirror serum KISS1 levels. Nonetheless, our study revealed lower levels of both placental KISS1 expression and plasma KISS1 expression in the placenta previa group compared to the control group. These consistent outcomes support the conclusion that KISS1 expression is diminished in patients with placenta previa.

There is a prevailing suggestion that KISS1 levels decrease in pregnant women experiencing preeclampsia and intrauterine growth restriction, conditions believed to be linked to placental development, when compared to uncomplicated pregnancies.^[17]^ Our study aligns with this notion, revealing lower levels of KISS1 both in plasma and placental tissue in patients with placenta previa. Armstrong et al have asserted that pregnancies associated with placental insufficiency exhibit lower maternal plasma KISS1 levels in the 2nd trimester.^[21]^ In a study conducted by Smets et al, it was reported that low plasma KISS1 levels in the first trimester were associated with low birth weight.^[22]^ Although the differences in KISS1 levels observed in these studies were moderate, the measurement of KISS1, while not serving as a standalone screening marker in preeclampsia and intrauterine growth restriction, was considered potentially useful when used in conjunction with other markers. Our study’s examination of patients with placenta previa suggests that determining KISS1 levels could play an effective role in diagnosing placenta previa.

Bilban et al have demonstrated that during the first trimester of pregnancy, placental KISS1, particularly Kisspeptin-10, has the capability to decrease collagenase activity and impede the migration and invasion of trophoblast cells.^[11]^ This interference with trophoblast invasion can result in a range of pregnancy abnormalities. Excessive trophoblastic invasion may lead to conditions like placenta acreata, increata, and percreata, while insufficient invasion could contribute to the occurrence of intrauterine growth restriction and preeclampsia.^[16]^ Research indicating decreased trophoblast invasion, along with increased placental KISS-1 mRNA and KISS1R expression in adverse pregnancy conditions such as preeclampsia and intrauterine growth restriction, emphasizes the regulatory role of Kisspeptin-10 in placental invasion.^[17]^ Our present study revealed a lower expression level of KISS1 in patients with placenta previa. This observation suggests that diminished KISS1 levels might have contributed to reduced pressure on trophoblastic invasion, potentially leading to the development of placenta previa due to excessive invasion. In this context, our study’s findings harmonize with existing literature and provide valuable insights into the etiology of placenta previa.

One of the limiting factors of the study is the absence of comprehensive investigations into angiogenesis, cell migration, and tissue modeling. These examinations may elucidate potential underlying mechanisms. Increasing the number of subjects and including cases with placenta previa findings in the first trimester could be used to increase the accuracy of the data. In animal studies where the kisspeptin gene is silenced, mechanism investigation may provide more useful results.

In this study, the diminished levels of KISS1 expression in patients with placenta previa is observed when compared to the control group. Both biochemical and genetic analyses consistently indicated significantly reduced KISS1 expression in individuals with placenta previa. Furthermore, our analysis of plasma levels revealed that KISS1 expression during the third trimester was lower than in the second trimester for patients with placenta previa. These collective findings suggest a lower expression of KISS1 in patients with placenta previa, potentially impairing their ability to regulate trophoblast invasion and leading to an abnormal course of placental invasion. Investigating markers associated with placental invasion, apoptotic and antiapoptotic processes may provide insights into the potential roles of KISS1 in placenta previa. Larger-scale studies measuring KISS1 in patients with placenta previa could shed light on its effectiveness in diagnosing the condition. Moreover, employing KISS1 replacement therapy in experimental animals with a placenta previa model may offer insights into KISS1’s potential roles in the etiology of placenta previa and its preventive effects. In conclusion, KISS1 may have a potential role in the etiology of the placenta previa. The further studies with larger scale may provide its possible role as a marker for placenta previa.

## Author contributions

**Conceptualization:** Yunus Katirci.

**Data curation:** Ayse Zehra Ozdemir.

**Formal analysis:** Yunus Katirci, Ayse Zehra Ozdemir.

**Investigation:** Yunus Katirci.

**Methodology:** Yunus Katirci, Adem Kocaman.

**Project administration:** Ayse Zehra Ozdemir.

**Resources:** Ayse Zehra Ozdemir.

**Software:** Adem Kocaman.

**Validation:** Yunus Katirci, Ayse Zehra Ozdemir.

**Visualization:** Adem Kocaman.

**Writing – original draft:** Yunus Katirci, Adem Kocaman.

**Writing – review & editing:** Yunus Katirci, Adem Kocaman, Ayse Zehra Ozdemir.
